# The accuracy of four commercial broth microdilution tests in the determination of the minimum inhibitory concentration of colistin

**DOI:** 10.1186/s12941-020-00383-x

**Published:** 2020-09-14

**Authors:** Erlangga Yusuf, Mireille van Westreenen, Wil Goessens, Peter Croughs

**Affiliations:** Department of Medical Microbiology and Infectious Diseases, Erasmus MC University Medical Center, Doctor Molewaterplein 40, 3015 GD Rotterdam, The Netherlands

**Keywords:** Colistin, Broth microdilution, Minimum inhibitory concentration, mcr-1, Cystic fibrosis

## Abstract

Colistin is considered as one of the last-resort antibiotics and reliable antimicrobial susceptibility testing is therefore crucial. The reference standard for AST according to EUCAST and CLSI is broth microdilution (BMD). However, BMD is labor intensive to perform. Commercial antimicrobial susceptibility tests derived from BMD method are available. We investigated the performance of four different commercial tests: Sensititre™, SensiTest™ Colistin, Micronaut MIC Strip Colistin and UMIC Colistin using 70 clinical isolates (half of them was deemed by VITEK2 as resistant), including isolates from cystic fibrosis patients and *mcr*-*1* bearing isolates. We used two reference standards: BMD and composite MIC as determined by all four tests. Sensititre™ had essential agreement (EA, defined as minimum inhibitory concentration within ± 1 dilution) of 87% and 89% compared to BMD and composite reference standard, respectively. For SensiTest™, the EA’s were 93% and 90%. For UMIC, 87% and 90%, and for Micronaut, 83% and 84%. All four tests demonstrated categorical agreement (CA) above 90%. CA for SensiTest™ and Micronaut was both 96%, UMIC 94%, and Sensititre™ 93%. All tests were reproducible as tested in two quality control isolates. In conclusion, in clinical isolates from a large referral center, the four commercial tests for determination of colistin minimum inhibitory concentrations showed acceptable performance.

## Introduction

Colistin is a polypeptide antibiotic that targets the outer membrane of Gram-negative bacteria [1]. It is considered as a last-resort antibiotic due to the increasing number of extensively drug-resistant bacteria and limited availability of new antimicrobial agents [2–5]. It is used in particular for treatment of infections caused by extensively drug-resistant (XDR) *Klebsiella pneumoniae*, *Pseudomonas aeruginosa* and *Acinetobacter baumanii* [5]. The proportion of microorganisms resistant to colistin is still relatively low at this moment but there is concern that it may increase in the future [5]. In a multicenter study in the US, the proportion of carbapenem resistant *K. pneumoniae* that were also colistin-resistant was 17% [6]. Reliable antimicrobial susceptibility testing (AST) for colistin is thus important, in order to prevent the patients receiving this nephrotoxic agent when it is unlikely to be effective.

Routine AST’s commonly used by microbiology laboratories include automated systems such as VITEK2. Yet, this system is not suitable for mucoid isolates that are often found in patients with cystic fibrosis. Other routine methods for colistin AST are gradient diffusion or disk diffusion. Yet, they are inappropriate because colistin molecules are large cationic molecules that diffuse poorly in the diffusion-based assays [7]. Another problem with gradient or disk diffusion is the need to use proper plastic ware since colistin can bind to polystyrene and influences the determination of colistin MIC [8]. Clinical and Laboratory Standard Institute (CLSI) and European Committee on Antimicrobial Susceptibility Testing (EUCAST) recommend broth microdilution (BMD) as the reference method [1]. Due to the restriction implemented by EUCAST and CLSI regarding AST for colistin, routine laboratories are now forced to introduce this laborious technique in their daily routine setting. Fortunately, several companies responded to this challenge, by bringing an easy-to use AST for colistin based on BMD method to the market [9, 10]. So far only a few studies have been published regarding the performance of commercial BMD colistin. Due to the low number of colistin resistant isolates in some setting, testing the commercial tests may be difficult. The study from EUCAST development laboratory included for example 75 samples and tested five commercial tests using isolates which originated from multiple sites in Europe [10] and did not include challenging isolates such as isolates originated from cystic fibrosis patients. Other studies used mixed isolates originated from human and veterinary samples [11] or only investigated one test [12].

In this study, we evaluated the performance of four commercially available BMD products for determination of colistin minimum inhibitory concentration (MIC) in comparison to the reference BMD method in isolates originated from a large referral center.

## Materials and methods

### Bacterial isolates

The microorganisms used in this study were isolated as part of standard care of patients admitted to Erasmus Medical Center University Hospital between 2009 and 2017. The isolates originated from various anatomical sites such as sputum, urine, tissues and blood. They were stored at -80◦C and identified to species level prior to microdilution test by using Microflex LT mass spectrometer (Bruker Daltonik GmbH, Bremen, Germany) with commercial database according to the manufacturer’s instructions. Half (n = 35) of the isolates were selected since they were deemed as colistin resistant by VITEK2 (bioMérieux SA, Marcy l’Etoile France) and were categorized as such according to European Committee of Antibiotic Susceptibility Testing (EUCAST) breakpoint, i.e. MIC of > 2 μg/mL [13]. Other isolates were selected to enrich the species variation or selected from specific patient groups.

Seventy isolates were thus used in this study, and consisted of 44 Enterobacterales (*Escherichia coli* (n = 19), *Klebsiella pneumoniae* (n = 14), *Enterobacter cloacae* complex (n = 3), *Enterobacter* spp. (n = 3), *Proteus hauseri* (n = 1), *Citrobacter freundii* (n = 1), *Kluyvera georgiana* (n = 1), *Klebsiella variicola* (n = 1), and *Serratia* spp. (n = 1)), and 26 non-fermentative Gram-negatives (*Pseudomonas aeruginosa* (n = 23), *Acinetobacter* spp. (n = 2), and *Achromobacter xylosoxidans* (n = 1)).

Thirteen isolates originated from patients with cystic fibrosis, eleven *P. aeruginosa,* one *K. variicola*, and one *A. xylosoxidans*. Among the Enterobacteriales, 17 (39%) were phenotypically positive for extended spectrum beta-lactamase (ESBL). ESBL was phenotypically detected using Combination Disc Test, i.e. disk diffusion by applying cefotaxime or ceftazidime alone, and in combination with clavulanic acid. ESBL was detected if the inhibition zone diameter was  ≥ 5 mm larger with clavulanic acid than without.

The collection was analyzed for the presence of *mcr*-*1* gene using PCR as described before [3], which was present in seven of the included isolates (five *E. coli,* one *K. georgiana*, and one *K. pneumoniae*).

Further, we included quality control (QC) strains *E. coli* ATCC 25922 (colistin susceptible), *E. coli* NCTC13846 (*mcr*-*1* positive), and *P. aeruginosa* ATCC 27853 (colistin susceptible).

### Reference standard

Reference MICs for colistin were determined using manual BMD, performed according to ISO standard 20776-1 [14]. MIC panels were prepared with pure colistin sulfate powder (Sigma–Aldrich, Illkirch, France) with two-fold dilutions in the range of 0.125 to 64 μg/mL in cation-adjusted MH broth (Becton–Dickinson, Sparks, MD, USA) in polystyrene plates (Greiner). Results were read after incubation in aerobic atmosphere at 35 ± 1 °C for 18 h.

BMD is a reference standard that is chosen by standardization institutes, but it might also prone to error as inherent variability can exist [15] and in this study because BMD uses polystyrene plastic. Therefore, to get an idea whether the performance of commercial MIC tests was robust, we created a composite reference standard using the MIC’s determined by the four commercial BMD tests. This approach could be considered as an alternative and/or complementary to the standard approach using BMD.

To investigate the robustness of the performance of the tests,. The MIC’s of the microorganism using this method are determined as follows. First, the range, i.e. the lowest and the highest MIC value from the four commercial tests was determined. When the difference between these two values was an even-fold number of dilutions, the middle dilution was set as the MIC of the microorganism. For example, when the lowest MIC was 8 µg/ml by one test, and the highest was 32 µg/ml by another test (twofold dilution difference), the MIC was set at 16 µg/ml. When the difference was uneven-fold, the middle two dilutions were chosen as the reference MIC values. For example, the lowest MIC of 8 µg/ml by one test, and the highest 64 µg/ml by another test (threefold dilution difference), the MIC was put at 16 µg/ml and 32 µg/ml.

### MIC determinations of colistin

MIC determinations for colistin were performed according to the manufacturers’ instructions by four commercially available BMD tests: YFRCOL Sensititre™, further referred to as Sensititre™ (Thermo Fisher Scientific, Cleveland, USA), SensiTest™ Colistin, further referred to as SensiTest™ (at present is re-branded as ComASP™ Colistin, Liofilchem, Roseto degli Abruzzi, Italy), Micronaut MIC Strip Colistin, further referred to as Micronaut (Merlin Diagnostika GmbH, Bornheim, Germany), and UMIC Colistin, further referred to as UMIC (Biocentric, Bandol, France).

Sensititre™ is a 96-wells plate containing colistin in two-fold dilution in the range of 0.125 to 128 µg/ml, allowing testing of eight isolates simultaneously. SensiTest™ has four panels, containing colistin in seven two-fold dilutions (0.25 to 16 µg/ml). Micronaut and UMIC are single isolate strips, containing freeze-dried colistin in 11 two-fold dilutions (0.0625 to 64 µg/ml).

One and the same suspension was prepared to inoculate these four systems (and the reference BMD method) in parallel. For Sensititre™, 10 μl of the 0.5 McF was suspended in 11 ml Mueller–Hinton (MH) broth provided by the manufacturer, of which 50 μl was inoculated in each well. For SensiTest™, the 0.5 McF solution was diluted 1:20 in saline, and 0.4 ml of this solution was added to a 3.6 ml tube of MH 2 broth provided by the manufacturer, and 100 µl of the mixed solution was dispensed into each well. For Micronaut, 50 µl of the 0.5 McF solution was added with 11 ml MH broth, and 100 µl of the solution was added in each well. For UMIC, the 0.5 McF suspension was directly diluted 1:200 in one of the MH2 tubes provided with the kit, of which 100 μL was added in each well of the unitary strip.

The results were read by unaided eye after incubation in aerobic atmosphere at 35 ± 1 °C for 18 ± 2 h and re-read after another 18 ± 2 h only if no growth was seen in the wells. Growth was considered when turbidity was present at the bottom of the well. The tests were considered as valid only if growth in the growth control was observed, and if no ‘skipped well’ occurred (i.e. no growth in a well but growth in a well with a higher colistin concentration).

### Statistical analysis

Accuracy, defined as the closeness of the result obtained with the commercial tests to the true value (BMD reference standard or the composite reference standard) [15] was determined by calculating essential agreement (EA) and categorical agreement (CA).

EA was defined as MIC determined by commercial tests that was within ± 1 dilution of the reference standard. CA was defined as total number of isolates tested using the commercial tests that yielded the same categorical interpretation as the reference standard according to EUCAST Breakpoint Tables version 9.0 (susceptible ≤ 2, resistant > 2 µg/ml) [13]. To cope with differences in concentration ranges between reference standard and commercial tests for the calculation of EA, the highest and lowest MIC concentration were considered as having essential agreement.

Very major error (VME) and major error (ME) proportions were calculated. VME was defined as resistant by reference BMD, but susceptible by commercial tests (false susceptible). ME was defined as susceptible by reference BMD but resistant by commercial tests. EA’s and CA’s were also determined by testing QC isolates *E. coli* ATCC 25922 (target MIC 0.5 to 1 µg/ml, range 0.25 to 2), *E. coli* NCTC13846 (target MIC 4 µg/ml, range 2 to 8), and *P. aeruginosa* ATCC 27853 (target MIC 1 to 2 µg/ml, range 0.5 to 4).

The reproducibility of the commercial tests was assessed by testing the latter two QC isolates three and six times, respectively. The tests were deemed as reproducible when the MIC’s were within ± onefold dilution.

## Results

### MIC characteristics of bacterial isolates according to BMD and commercial tests

**Fig. 1:**
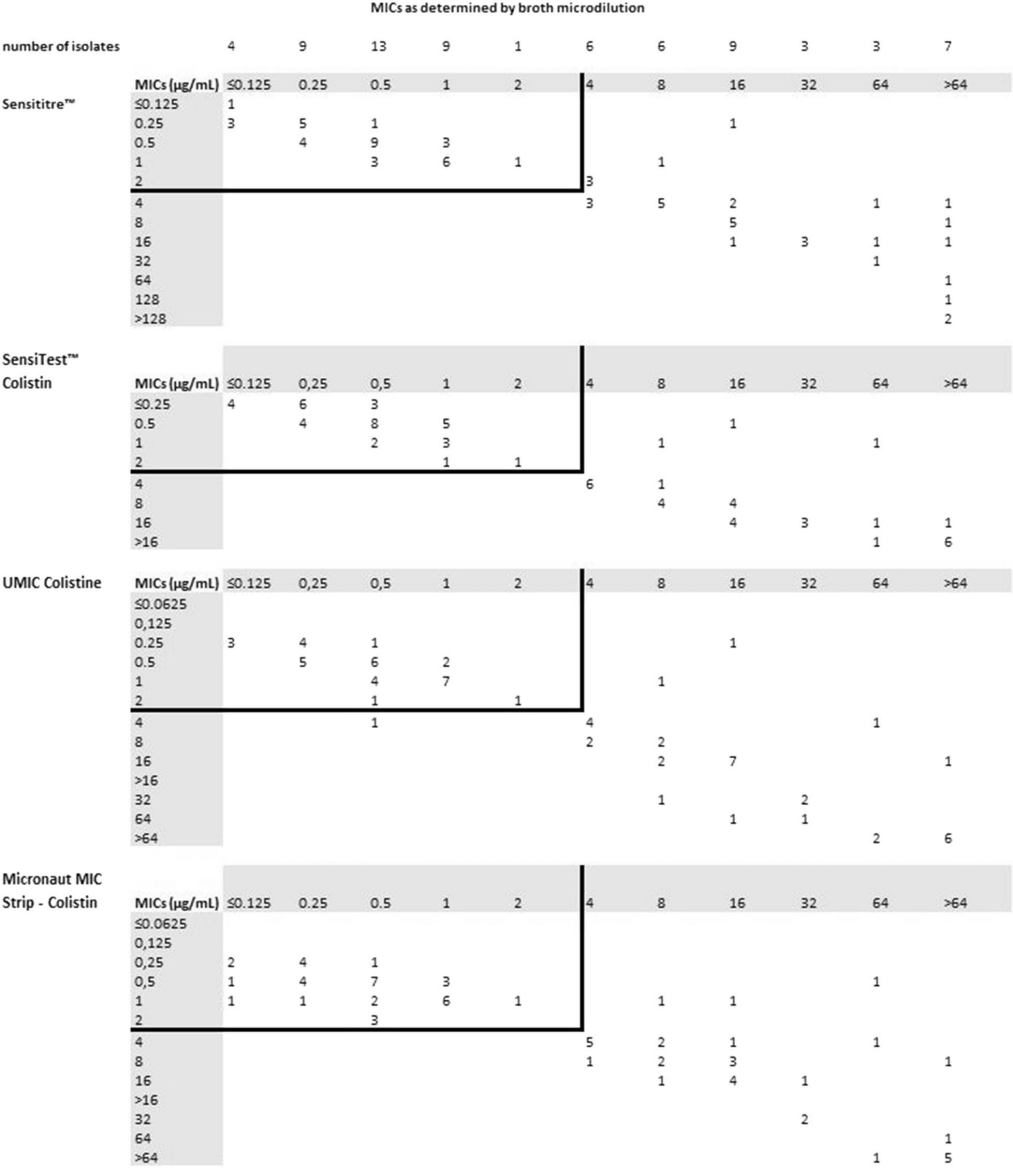
The MIC distribution of the included isolates as determined by the four commercial tests and broth microdilution reference standard

The MIC distribution of the included isolates as determined by the four commercial tests are presented in Fig. 1. In two isolates, Sensititre™ measured a MIC above its upper MIC dilution limit (> 128 µg/ml) and in one isolate at or below its lower MIC dilution limit (≤ 0.125 µg/ml). SensiTest™ measured seven isolates above its upper dilution limit (> 16 µg/ml), and thirteen at or below lower level of dilution limit (≤ 0.25 µg/ml). By UMIC and Micronaut, with similar dilution range, eight and six isolates respectively were measured above upper dilution limit (MIC > 64 µg/ml) and no isolates had a MIC ≤ 0.0625 µg/ml.In more than the half of the isolates, the MIC’s determined by the four commercial tests were either the same (n = 17, 24%), or within ± 1 dilution (n = 28, 40%). Two isolates had MICs above upper MIC dilution limits by all four tests. A difference of ± 2 dilutions occurred 13 times (19%), and ± 3 or more in 10 isolates (14%). The maximum difference among the commercial tests was 8 dilutions, in a *P. aeruginosa* (MICs in µg/ml according to Sensititre™ 4, SensiTest™ > 16, UMIC > 64, Micronaut MIC Strip 0.5).

The MIC could not be determined in one *P. aeruginosa* isolate by all four tests after 18 + 2 h of incubation and in another *P. aeruginosa* isolate by all tests except Sensititre™ because of lack of growth. In four isolates, a skipped well occurred in UMIC and Micronaut at 18 ± 2 h of incubation. In all cases, except for one *Acinetobacter* spp. using UMIC, MIC’s could be read after another 18 ± 2 h of incubation.

### Performance of commercial MIC colistin tests

The commercial tests had EA’s of above 80% compared to BMD reference standard (Table 1), SensiTest™ showed the highest EA (92.8%, 65/70) and Micronaut showed the lowest EA (82.9%, 58/70) and the EA’s remained above 80% when the alternative composite reference standard was used. Compared with Enterobacteriales, the performance of the commercial tests on non-fermenters was lower; the EAs of UMIC and Micronaut were lower than 80% (76.9%, 20/26 and 69.2% 18/26 respectively). The performance of commercial tests for non-fermenters was increased when composite reference standard was used.

**Table 1:** Essential Agreements of commercial MIC tests compared to reference standard Broth Microdilution, n (%)

Isolates	n	Sensititre™		SensiTest™ colistin		UMIC colistine		Micronaut MIC strip–colistin	
		vs. BMD	vs. composite reference	vs. BMD	vs. composite reference	vs. BMD	vs. composite reference	vs. BMD	vs. composite reference
Total	70	61 (87.1)	62 (88.6)	65 (92.8)	63 (90.0)	61 (87.1)^a^	62 (88.6)^a^	58 (82.9)	59 (84.3)
*Enterobacteriales*	44	39 (88.6)	38 (86.4)	42 (95.5)	40 (90.9)	41 (93.2)	38 (86.4)	40 (90.9)	38 (86.4)
*Escherichia coli*	19	19 (100)	19 (100)	19 (100)	18 (94.7)	19 (100)	19 (100)	19 (100)	19 (100)
*Klebsiella pneumoniae*	14	11 (78.6)	12 (85.7)	13 (92.9)	14 (100)	13 (92.9)	13 (92.9)	12 (85.7)	12 (85.7)
*Enterobacter cloacae* complex	3	1 (33.3)	3 (100)	3 (100)	3 (100)	3 (100)	2 (66.7)	2 (66.7)	2 (66.7)
*Enterobacter* spp.	3	3 (100)	1 (33.3)	3 (100)	1 (33.3)	3 (100)	1 (33.3)	2 (66.7)	1 (33.3)
*Proteus hauseri*	1	1 (100)	1 (100)	0 (0)	1 (100)	1 (100)	0 (0)	1 (100)	0 (0)
*Citrobacter freundii*	1	1 (100)	1 (100)	1 (100)	1 (100)	0 (0)	1 (100)	1 (100)	1 (100)
*Kluyvera georgiana*	1	1 (100)	0 (0)	1 (100)	1 (100)	0 (0)	0 (0)	1 (100)	1 (100)
*Klebsiella variicola*	1	1 (100)	1 (100)	1 (100)	1 (100)	1 (100)	1 (100)	1 (100)	1 (100)
*Serratia* spp.	1	1 (100)	0 (0)	1 (100)	0 (0)	1 (100)	1 (100)	1 (100)	1 (100)
*Non fermentative Gram-negatives*	26	22 (84.6)	24 (92.3)	23 (88.5)	23 (88.5)	20 (76.9)	24 (92.3)	18 (69.2)	21 (80.8)
*Pseudomonas aeruginosa*	23	20 (86.9)	22 (95.7)	20 (87.0)	20 (87.0)	18 (78.6)	22 (95.7)	17 (73.9)	20 (87.0)
*Acinetobacter* spp.	2	2 (100)	1 (50)	2 (100)	2 (100)	1 (50)^a^	1 (50)^a^	1 (50)	1 (50)
*Achromobacter xylosoxidans*	1	0 (0)	1 (100)	1 (100)	1 (100)	1 (100)	1 (100)	0 (0)	

^a^ No growth counted as disagreement

All four tests demonstrated a CA above 90%, with SensiTest™ and Micronaut both 95.7% (67/70), UMIC 94.3% (66/70), and Sensititre™ 92.9% (65/70) (Table 2). The EA’s using commercial MIC tests for colistin were considerably better than VITEK2.

**Table 2:** Categorical Agreements of commercial MIC tests compared to reference standard Broth Microdilution, n (%)

	Categorical agreement, n (%)	Very major error, n (%)	Major error, n (%)
Sensititre™	65 (92.9)	5 (14.7)	0 (0)
SensiTest™	67 (95.7)	3 (8.8)	0 (0)
UMIC	66 (94.3)	2 (5.9) ^a^	1 (0) ^a^
Micronaut	67 (95.7)	3 (8.8)	0 (0)
VITEK2 (n = 56)	47 (83.9)	3 (8.8)	6 (10.7)

^a^ No growth in one isolate

Two isolates were consistently deemed as VME across the four commercial tests, a *P. aeruginosa* (BMD MIC 8 µg/ml, harbouring *bla*_VIM-1_) and a *K. pneumoniae* (MIC according to BMD 16 µg/ml) (Table 3). Next to these isolates, Sensititre™ showed false susceptibility (VME) in one *Acinetobacter* spp. (MIC 2; BMD MIC 4 µg/ml), and two *E. coli*’s (both MIC 2; BMD MIC 4 µg/ml and both were positive for extended spectrum beta-lactamases). SensiTest™ showed a third VME in one *P. aeruginosa* from a cystic fibrosis patient (MIC 1; BMD MIC 8 µg/ml). Micronaut also showed a third VME in one *P. aeruginosa* (MIC 0.5; BMD MIC 64 µg/ml, isolated from a CF patient).

**Table 3:** Isolates with VME or ME (by any commercial BMD test) and their corresponding MICs (µg/ml)

Isolates	BMD reference standard^a^	Sensititre™ (VME n = 5, ME n = 0)	SensiTest™ (VME n = 3, ME = 0)	UMIC (VME n = 2, ME = 1)	Micronaut (VME n = 3, ME = 0)
*Pseudomonas aeruginosa*	0.5	0.5	≤ 0.25	4	2
*Pseudomonas aeruginosa*	8	1	1	1	1
*Pseudomonas aeruginosa*	64	4	> 16	> 64	0,5
*Pseudomonas aeruginosa*	64	16	1	4	4
*Acinetobacter junii*	4	2	4	4	4
*Escherichia coli*	4	2	4	8	4
*Escherichia coli*	4	2	4	4	4
*Klebsiella pneumoniae*	16	0.25	0.5	0.25	1

*VME* very major error, *ME* major error

^a^BMD: broth microdilution

### Performance of the commercial tests in cystic fibrosis and mcr-1 gene harboring isolates

The EA’s of the commercial tests in the 13 isolates originating from cystic fibrosis patients were 76.9% (Sensititre™; 10/13), 92.3% (SensiTest™; 12/13), 84.6% (UMIC; 11/13), and 69.2% (Micronaut; 9/13). The CA’s were 100% for Sensititre™ and 92.3% for the other three tests.

All seven isolates harboring *mcr*-*1* gene were deemed as resistant by the four commercial tests, except for one, which was tested susceptible by Sensititre™ (MIC 2 µg/ml; BMD MIC 4 µg/ml). The MIC of one isolate according to Sensititre™ (4 µg/ml) was two dilutions lower than BMD (16 µg/ml). The remaining five isolates, had a MIC of 4 µg/ml, which was in agreement with BMD. SensiTest™ and Micronaut showed essential agreement for all seven *mcr*-*1* gene isolates. UMIC showed essential agreement in six out of the seven isolates; in one isolate the MIC was two dilutions higher than BMD (8 µg/ml).

### Reproducibility

All commercial tests showed 100% reproducibility. Sensititre™ showed exactly the same MIC for *E. coli* NCTC 13846 and for *P. aeruginosa* ATCC 27853 in all three and six reproductions respectively.

In *E. coli* NCTC 13846 SensiTest™, UMIC and Micronaut showed exactly the same MIC in two out of three repeats. SensiTest™ showed exactly the same MIC in five out of six reproductions of *P. aeruginosa* ATCC 27853, by UMIC MICs for this isolate were the same six times, and by Micronaut there was one doubling dilution difference three out of the six repeats.

## Discussion

In this study we investigated the performance of four commercial tests to determine MIC of colistin. To the best of our knowledge, only a few studies have evaluated these tests. Our study evaluated these four tests in clinical isolates. Also included were isolates from cystic fibrosis patients, which are often mucoid and cannot be tested using VITEK2.

Our results showed that the CA of the four commercial tests were high, i.e. ≥ 90%. These high CA’s are comparable with a study from EUCAST Development laboratory using 75 international isolates where Sensititre™, SensiTest™, UMIC and Micronaut were also tested [10]. The categorical disagreement in Sensititre™ was caused mainly by two isolates with the MIC around the breakpoint, where Sensititre™ showed a lower MIC than the reference. This tendency was also found in the EUCAST study [10] and can be explained by the absence of intermediate (‘I’) category in EUCAST and CLSI breakpoint tables, that is normally used as a buffer zone for possible technical errors.

Jayol et al. evaluated Sensititre™ and UMIC on a collection of 185 Gram-negative isolates, of which 39 were non-fermenters, and found VME of 3.0% and 11.3%, respectively [16]. In our study, we found VME of 14.7% for Sensititre™ and 5.9% for UMIC. The difference might be due to the selection of isolates. This is also the reason why a hard cut-off of acceptable percentages of VME’s and ME’s as used by standardization bodies might be not appropriate to be used in this specific study. In our study, we included many challenging isolates and it might increase the number of VME’s and ME’s. This emphasizes the need of evaluating various commercial tests using different isolates with various difficulties (i.e. mucoid/not mucoid, antimicrobial resistance profile) in several geographical regions.

The high EA between the commercial tests and BMD can and should be expected since they used comparable AST method and the difference would be merely based on the type of colistin and type of materials used for the wells. It is known that colistin binds to the polystyrene and efforts have been made to prevent this. Interestingly in our study, we found that the EA’s of the commercial tests when composite reference standard was used for non-fermenters were better than with BMD as reference standard. This difference was not noticed on Enterobacteriales. It suggests that for non-fermenters the MICs of commercial tests are more equal and less comparable to the BMD reference test.

We also found in our study that Micronaut had a rather low% EA (69.2%) in isolates from cystic fibrosis patients. Accurate susceptibility testing of *P. aeruginosa* from cystic fibrosis patients is often challenging because the organisms are often mucoid and slowly growing [17]. To the best of our knowledge, apart for Sensititre™ [12] none of the published studies regarding commercial tests for colistin mentioned isolates from cystic fibrosis patients. The% EA in their study increased, from 82% after 24 h incubation to 95% after 48 h incubation.

Since the MICs of the resistant *mcr*-*1* harboring isolates do not necessarily need to be increased, the commercial tests are not optimal for screening of the presence of this gene for the infection prevention purposes. The commercial tests in this study did allow detection of the expression of these genes.

From a practical point of view, each of the different commercial tests may have its advantages and disadvantages. Sensititre™ and Sensitest™ are designed for testing respectively eight and four isolates simultaneously. These tests may be applicable to be used for testing in a batch in a large laboratory. For Sensititre™, there is also a possibility for automatic inoculation and the use of an automated reader. The single strip tests by UMIC and Micronaut can be used for immediate testing without waiting for enough isolates to be tested simultaneously. When no MIC can be read after 18 ± 2 h of incubation, another incubation is needed. For UMIC and Micronaut, care should be taken that wells do not desiccate, for which a special box is available.

In rare occasions, skipped-well occurred in this study. We do not have a clear explanation to explain this phenomenon. We can only speculate that pipetting error, contact with polystyrene plastics may cause this problem.

The strength of our study is that we also included challenging isolates such as isolates from cystic fibrosis patients. Our study adds to the body of literature where isolates originated from other parts of the world. The results of our study can be used for other labs in order to organize the flow of their lab regarding colistin AST. In our lab, due to the findings described in this study, we confirm the resistant VITEK2 AST results using commercial colistin MIC methods.

In conclusion, the four commercial colistin MIC methods investigated in this study showed acceptable performance when they were compared with reference BMD standard.

## Data Availability

The datasets during and/or analysed during the current study available from the corresponding author on reasonable request.
